# Complex Atheromatosis of the Aortic Arch in Cerebral Infarction

**DOI:** 10.2174/157340310791658712

**Published:** 2010-08

**Authors:** Ramón Pujadas Capmany, Montserrat Oliveras Ibañez, Xavier Jané Pesquer

**Affiliations:** Department of Cardiology, Hospital Universitari del Sagrat Cor, Address: Viladomat 288, E-08027 Barcelona, Spain

**Keywords:** Stroke, aortic arch atheroma, complex aortic plaques.

## Abstract

In many stroke patients it is not possible to establish the etiology of stroke. However, in the last two decades, the use of transesophageal echocardiography in patients with stroke of uncertain etiology reveals atherosclerotic plaques in the aortic arch, which often protrude into the lumen and have mobile components in a high percentage of cases. Several autopsy series and retrospective studies of cases and controls have shown an association between aortic arch atheroma and arterial embolism, which was later confirmed by prospectively designed studies. The association with ischemic stroke was particularly strong when atheromas were located proximal to the ostium of the left subclavian artery, when the plaque was ≥ 4 mm thick and particularly when mobile components are present. In these cases, aspirin might not prevent adequately new arterial ischemic events especially stroke. Here we review the evidence of aortic arch atheroma as an independent risk factor for stroke and arterial embolism, including clinical and pathological data on atherosclerosis of the thoracic aorta as an embolic source. In addition, the impact of complex plaques (≥ 4 mm thick, or with mobile components) on increasing the risk of stroke is also reviewed. In non-randomized retrospective studies anticoagulation was superior to antiplatelet therapy in patients with stroke and aortic arch plaques with mobile components. In a retrospective case-control study, statins significantly reduced the relative risk of new vascular events. However, given the limited data available and its retrospective nature, randomized prospective studies are needed to establish the optimal secondary prevention therapeutic regimens in these high risk patients.

## INTRODUCTION

With the progressive increase in life expectancy in Western populations, the incidence of cerebral ischemic diseases has become a direct cause of death of 10% of the general population. In addition, another 10% of deaths are related to the consequences and complications following cerebral infarction. 

In the first half of the 20th century, it was considered that the majority of cerebral infarctions were due to an episode of vasospasm, but currently this mechanism is not considered of great importance. Basically, this view has changed since 1951, when C. Miller Fisher described the trascendental etiological role of artery-artery embolism from carotid atherosclerotic plaques [1]. Subsequently, it has been shown that approximately 25% of cerebral ischemic events are due to cardioembolism [2] with atrial fibrillation involved in two thirds of these patients [2], which is a relevant finding to establish a correct diagnosis given the need of anticoagulation and the increasing trend to start anticoagulants very early [3,4]. Because of the importance of both etiologies, most current protocols for assessing cerebral ischemia include specific radioimaging studies of the cranial arterial system (Doppler ultrasound evaluation of supra-aortic arteries, optionally in association with angiography or magnetic resonance angiography), as well as cardiological studies including electrocardiogram, transthoracic echocardiogram (TTE), and Holter electrocardiographic monitoring if paroxysmal atrial arrhythmias are suspected. Despite the use of these diagnostic studies together with routine screening of possible coagulopathy or systemic diseases, and the use of transcranial Doppler sonography with the Valsalva maneuver to rule out the presence of patent foramen ovale with right-to-left shunt, it is not possible to establish a definitive etiologic diagnosis in 10-40% of ischemic stroke patients [5-8].

The management of patients with cerebral infarction focuses on measures that will reduce the risk of stroke recurrence. These secondary prevention strategies include quitting smoking, the administration of aspirin, antihypertensive and lipid-lowering treatments to reduce blood pressure and cholesterol (even when in the "normal" range), surgical or percutaneous interventions on significant stenosis of the symptomatic carotid artery, and anticoagulation in the case of cardioembolism. However, in the context of secondary prevention it is essential to reach an etiological diagnosis in order to prescribe the optimal measures for each individual patient, and in this respect, stroke of uncertain etiology remains a great clinical challenge. Between the heart and the carotid and vertebral arteries there is the aortic arch, an area of difficult access for diagnostic techniques commonly used in daily practice. In recent years data have been emerging on the association of aortic arch atheromas and cerebral infraction, in particular, when no obvious etiology could be found. Thus, there is an increasingly perception of this particular vascular territory as the “missing link” responsible for most of the cerebral infarctions of undetermined cause, and it is particularly evident in case of recurrent ischemic cerebral events despite the use of adequate general treatment measures.

## AORTIC ARCH ATHEROMATOSIS

Aortic arch atheroma can be seen in some young adults but its incidence and severity increases with age [9, 10]. This condition is associated with smoking, hypercholesterolemia, hypertension, diabetes, male sex, and elevated plasma levels of fibrinogen and homocysteine [11, 12]. Its natural history is very variable and not always progressive. In three echographic studies of the morphological changes of aortic arch atheromas at 6-12 months of follow-up, plaque thickness had increased in 8%, 23% and 37% of lesions, but it improved in 2%, 10% and 22%, and remained unchanged in 41%, 67% and 90% of the cases, respectively [10, 13, 14]. These data suggest that atheromatosis is a dynamic process and that although plaque regression is possible, the most likely clinical course is slow progression.

In the first half of the past century, some pathologists suggested that "eroded atherosclerotic plaques" could result in embolic arterial occlusions [15], and in the 60s some case reports supporting this concept were published [16, 17]. However, these findings could not be assessed *in vivo* until the introduction of transesophageal echocardiography (TEE) in the 80s, --a highly complex imaging technique minimally invasive, with a very low risk of complications--, which permits to directly visualize some cardiac and vascular structures previously inaccessible (e.g., the aortic arch). In 1990, Tunick and Kronzon [18] reported the cases of three patients of 68, 70 and 77 years of age, respectively, presenting with acute cerebral ischemia (in one case associated to peripheral arterial embolism), in whom TEE documented large atheromatous plaques which protruded strikingly into the aortic arch lumen and had obvious mobile components, the probable etiology of embolism being attributed to them.

## PREVALENCE OF AORTIC ARCH ATHEROMA

In a series of 2700 autopsies of patients between 15 and 64 years of age who died in New Orleans between 1960 and 1968 by external violence or natural causes other than coronary heart disease, stroke, diabetes or hypertension, the prevalence of aortic plaques increased with age from 0.4% at 15-24 years of age up to 33% at 55-64 years [19]. The Stroke Prevention Assessment of Risk in the Community (SPARC) study [20], a random sample of 581 people over age of 44 years in Olmstead County Minnesota, was evaluated. All participants had TEE and atheroma was identified in 51.3% of patients, been characterized as severe atheroma in 7.6% (plaque thickness ≥ 4 mm, ulcerated, or with mobile component). In this random population, the prevalence of complex aortic arch atheroma increased with age, with severe atheroma documented in more than 20% of patients older than 70 years.

Davila-Roman *et al.* [9] analyzed perioperative epiaortic ultrasound examinations in 1200 patients of more than 49 years of age having open heart surgery (88% of them having coronary artery bypass grafting). In this series, complex plaques (plaque thickness ≥ 3 mm, ulcerated or mobile components) were found in 19.3% of patients, and its prevalence increased with age (from 9.6% in the range between 50-60 years to 32.6% in patients over 80 years). The fact that in this series the prevalence of complex plaques was greater than that recorded in the SPARC study [20] (19.3% *vs *7.6%) could be attributed both to the selection of patients (88% with coronary artery disease) and to the inclusion as “complex plaque” of atheromas between 3 and 3.9 mm of plaque thickness.

## PREVALENCE OF AORTIC ARCH ATHEROMA IN PATIENTS WITH STROKE

### Autopsy Studies

In 1992, Amarenco *et al.* [21] published a review of 500 consecutive autopsies in patients with neurological diseases. Ulcerated plaques were documented in the aortic arch in 62 of 239 (26%) patients who died from stroke but in only 13 of 261 (5%) patients who died from other neurological diseases (*P* < 0.001). Interestingly, such plaques were seen in 17 of 28 (61%) patients who died from cerebral infarction of unknown etiology as compared to only 34 of 155 (22%) patients with an identifiable cause of ischemic stroke (*P* <0.001).

In a series of 120 consecutive hospital autopsies, Khatibzadeh *et al.* [22] found 36 cases with pathological evidence of cerebral infarction. In a multiple linear regression analysis, stroke was associated with atrial fibrillation (hazard ratio [HR] = 3.5; 95% confidence interval [CI] 1.1- 9.9), ipsilateral carotid stenosis greater than 75% (HR = 11.7, 95% CI 3.1-45.3), and complex plaques in the aortic arch (ulcerated, with mural thrombosis or atheromatous debris) (HR = 5.8, 95% CI 1.1–31.7).

### Non-Controlled Ultrasound Studies

As previously mentioned, Tunick and Kronzon [18], in 1990, reported three patients with acute cerebral ischemia in whom assessment of the aortic arch by TEE showed large plaques with mobile components protruding into the lumen, and embolism was likely attributed to these lesions. In the same year, Pop *et al.* [23] reported TEE findings in 72 consecutive patients with stroke or transient cerebral ischemia, documenting aortic arch plaques in 32 (44.4%). In similar studies conducted in patients with cerebral ischemia, the prevalence of aortic arch plaques ranged between 14% and 42% [24, 25]; in another study, plaques with endoluminal mobile components were documented in 4 of 183 patients [26].

### Retrospective Case-Control Ultrasound Studies

Several retrospective studies of cases and controls found a significant association between stroke or peripheral embolism and aortic arch atheromas. In three studies based on TEE [27-29], the prevalence of aortic arch atheroma in patients with previous embolism ranged between 21% and 27%, whereas ranged between 4% and 13 % in controls. The multivariate analysis showed that patients with aortic arch atheroma had a significantly higher prevalence of previous stroke (odds ratio [OR] between 3.2 and 8.2). On the other hand, in a study of 1200 perioperative epiaortic ultrasound studies in patients undergoing open heart surgery (coronary bypass surgery in 88% of the cases), Davila-Roman *et al.* [9] found that the prevalence of aortic arch atheroma was significantly higher (28%) in patients with previous stroke or transient cerebral ischemia than in those without history of cerebrovascular events (18%). 

Nine studies [29-37] examined the prevalence of aortic arch atheroma in patients undergoing TEE for the evaluation of stroke or peripheral embolism, including patients referred for echocardiography for other reasons (controls). In these studies, when TEE showed the presence of aortic arch atheroma the OR of previous stroke or peripheral embolism ranged between 1.92 and 16.52.

### Plaque Morphology and Embolic Risk in Retrospective Case-Control Studies

The relationship between aortic arch atheroma and stroke is even more evident when the morphologic characteristics of the plaques are assessed. The most significant association relies on the presence of endovascular mobile components, its pedunculated appearance, plaque ulceration, and the highest degrees of plaque thickness.

Kazui *et al.* [38] performed TEE in 62 patients with lacunar infarction and in 202 healthy controls and found aortic arch atheromas ≥ 5 mm thick in 20% of lacunar stroke patients compared with only in 4% in controls (OR = 5.94, 95% CI 2.30-15.32). Karalis *et al.* [39] reviewed 556 unselected TEE studies and documented previous stroke or peripheral embolism in 11 of 36 (30.6%) patients with aortic arch complex plaques (≥ 5 mm thick) in comparison with 4 of 100 (4%) age-matched controls without complex aortic arch plaques. In this study, plaque morphology also seemed to be important, with previous stroke or arterial embolism in 8 of 11 (72.7%) patients with pedunculated mobile atheromas and in only 3 of 25 (12%) patients with immobile plaques. In a case-control study, Tunick *et al.* [27] evaluated 122 patients referred to TEE for evaluation of stroke, transient cerebral ischemia, or unexplained arterial embolism and 122 controls matched for age and sex referred for other reasons. Complex aortic arch atheroma (≥ 5 mm in plaque thickness or presence of mobile components) was found in 33 of 122 (27%) cases compared with in 11 of 122 (9%) controls (OR = 3.74, 95% CI 1.79-7.82). Mobile atheroma was observed in 11 of 122 cases (9%) and in none of patients in the control group.

In five patients with mobile lesions it was demostrated at the time of surgery that the moving images corresponded to thrombi attached to ulcerated or fissured atherosclerotic plaques [35, 40, 41]. Likewise, it has been described the disappearance of these mobile lesions after anticoagulation [42, 43] and thrombolysis [44]. The most remarkable common finding in all these observational or retrospective studies in patients with stroke or peripheral embolism is the high prevalence of atherosclerosis of the aortic arch (between 20% and 30%), which results of similar (or even greater) magnitude than the prevalence of carotid artery stenosis or atrial fibrillation in patients with stroke [5, 28, 45].

Besides plaque thickness and the presence of moving components, plaque ulceration also confers poor prognosis. In an autopsy study [21], ulcerated aortic arch plaques were found in 61% of 28 patients with stroke of uncertain cause as compared with in only 22% of 155 stroke patients with an apparently obvious etiology of stroke (*P* < 0.001); after adjusting by covariates, the prevalence of plaque ulceration was 57.8% and 20.2%, respectively (adjusted OR = 5.7, 95% CI 2.4-13.6, *P* < 0.001). In a TEE study [32] the presence of plaque ulceration (≥ 2 mm) was highly correlated with cryptogenic stroke. Thus, ulcerated plaques were found in 9 of 23 patients (39.1%) with stroke of unknown etiology as compared with 2 of 26 patients (7.7%) with an identifiable cause of stroke, and in only 4 of 57 patients (7%) with no clinical history of stroke.

Despite the strong association between atheroma of the aortic arch and embolism, which is particularly evident in the case of thickest plaques and those with moving components and/or ulceration, causality could not be definitely established because these data were basically descriptive or had been obtained retrospectively.

## AORTIC ARCH ATHEROMA AS A PROBABLE CAUSE OF STROKE

### Causality and Anatomical Location of Atheromas

The SPARC study [45] included a random population of 585 patients at high risk of vascular events in which TEE data were obtained. The mean age was 66.9 years, and aortic plaques were detected in at least one of the three aortic segments (ascending, arch, and descending) in 252 patients (43.1%). In 44 cases (7.5%), plaques were morphologically complex (≥ 4 mm thick, ulcerated, or with mobile components). The prevalence of simple and complex plaques in the ascending aorta was 8.4% and 0.2%, respectively, but in the aortic arch the prevalence increased to 31.0% and 2.2%, and in the descending aorta to 43.7% and 6.0%. Therefore, the great majority of simple (90.1%) and complex (97.6%) aortic arch atheromas are located distally to the innominate artery. It is particularly interesting that only 18% of embolic events involved the right brain (versus 82% the left brain or peripheral arteries) [27], and since it is not expected for atheromas located distal to the innominate artery to develop retrograde embolization to the right brain (except in the unlikely event of a severe aortic valve insufficiency), this observation also supports the hypothesis of causality for aortic arch atheroma in the pathogenesis of stroke and peripheral arterial embolism.

### Case-Control Studies

Prospective studies can overcome most of the limitations inherent to retrospective series (Table ** 1**). In 1994, Amarenco *et al.* [46] reported a prospective case-control study which included 250 consecutive patients over 60 years of age referred for TEE during hospitalization for stroke, and 250 consecutive controls over 60 years of age without previous stroke referred to TEE for valvular heart disease, ischemic heart disease, atrial fibrillation, or suspected endocarditis. The stroke patients were significantly older than controls (mean age 76 vs 72 years) and showed a higher occurrence of hypertension (67% vs 35%), hypercholesterolemia (31% vs 24%), cigarette smoking (41% vs 32 %), and diabetes (18% vs 12%). Patients with cerebral infarction had a higher prevalence of atheroma 1–3.9 mm thickness in the ascending aorta or the aortic arch (46% vs 22%, OR = 4.4 after adjusting for cardiovascular risk factors, *P *< 0.001). In this study the prevalence of plaques ≥ 4 mm thick located in the ascending aorta or the aortic arch was also significantly higher in stroke patients than in controls (14.4% vs 2.0%, *P* <0.001), and after adjustment for cardiovascular risk factors, patients with plaques ≥ 4 mm thick showed a high risk for stroke (OR = 9.1). Moreover, the HR for cerebral infarction increased with plaque thickness, from 1.0 (no risk) for athermas 0–1 mm thick to 4.4 (95% CI 2.8–6.8) for atheromas 1–3.9 mm and to 13.8 (95% CI 5.2–36.1) for atheromas ≥ 4 mm. In the subgroup of 78 patients with stroke of undetermined cause, the prevalence of plaques ≥ 4 mm thick was 28.2%, compared with only 8.1% in 172 patients with cerebral infarction in which the etiology of stroke was identified (OR = 4.7, 95% CI 2.2-10.1, *P* < 0.001).

Jones *et al.* [28] reported a prospective case-control study that included patients of any age with stroke or transient ischemic attack referred for TEE and ambulatory controls. Patients had a higher prevalence of atrial fibrillation, hypertension, smoking, diabetes, and carotid artery stenosis. In stroke patients, the adjusted OR for non-complex atheroma (< 5 mm plaque thickness) was 2.3 (95% IC 1.2–2.4) but when the analysis was restricted to complex plaques (≥ 5 mm thick, ulcerated, or mobile components) the adjusted OR increased to 7.1 (95% CI 2.7-18.4).

### Follow-Up Studies: Plaque Thickness, Morphology, and Embolic Risk

In the initial prospective studies (Table ** 1**), plaque thickness ≥ 5 mm was used as the main criterion to define complex atheroma [27, 39]. In 1994, Tunick *et al.* [47] reported a prospective case-control study involving 42 patients with complex plaques (≥ 5 mm thick or presence of mobile components) without evidence of other possible causes of embolism. These patients were selected from 521 consecutive patients undergoing TEE over 1-year period. A group of 42 controls with comparable age, sex, and prevalence of hypertension referred for TEE without previous embolism, complex aortic plaques, carotid arterial disease or heart diseases associated with embolic risk was included. During an average follow-up period of 14 months, 14 patients with complex atheromas had vascular events (33.3%) compared with only 3 controls (7.1%). In the multivariate analysis, complex atheroma was the only independent risk factor for vascular events (HR = 4.6, 95% CI 1.1-18.9). Similarly, in the study of Mitush *et al.* [48] during a median follow-up of 16 months patients with complex aortic arch plaques in the baseline TEE (≥ 5 mm thick, or mobile components) had a prevalence of systemic embolism of 18.9 per 100 patient-years (11 of 47) compared with only 4.6 per 100 patient-years (8 of 136) in controls without complex plaques.

The largest study was the French Study of Aortic Plaque in Stroke (FSAPS) Group [49], in which 331 consecutive stroke patients aged ≥ 60 years were followed for 2 to 4 years. All patients had an initial TEE to assess the presence of atherosclerotic plaques in the aortic arch proximal to the ostium of the left subclavian artery. Patients were stratified into three groups depending on aortic plaque thickness (<1 mm, 1 to 3.9 mm, and ≥ 4 mm). At follow-up, the group of patients with plaques ≥ 4 mm showed an incidence of 11.9 strokes per 100 patient-years as compared with 3.5 per 100 patient-years in patients with plaques between 1 and 3.9 mm, and 2.8 per 100 patient-years in those with plaques < 1 mm (*P* <0.001). The incidence of any arterial vascular event in the three study groups was 26.0, 9,1, and 5.9 per 100 patient-years, respectively (*P* < 0.001). After adjustment for the presence of carotid stenosis, atrial fibrillation, peripheral arterial disease, and other risk factors, plaque thickness ≥ 4 mm was an independent predictor of both recurrent stroke (relative risk [RR = 3.8, 95% CI 1.8–7.8, *P* = 0.0012) and any new arterial vascular event (RR = 3.5, 95% CI 2.1–5.9, *P* < 0.001). The presence of plaques ≥ 4 mm thick in the descending thoracic aorta (which theoretically cannot embolize retrogradely to the brain) was associated with an OR for ratio for stroke of 1.5, whereas identical plaques located proximal to the ostium of the left subclavian artery were associated with an OR of 13.8. This finding also supports the concept of causality of aortic arch complex plaques and cerebral embolism.

Since the FSAPS study [49], it has been accepted that the two main criteria for embolic risk associated with aortic arch atheromas are plaque thickness ≥ 4 mm and the presence of mobile components. During three consecutive years Ferrari *et al.* [37] performed 1116 TEE and observed aortic arch atheromas in 139 patients (85% of whom had been referred by an embolic event). These patients were followed for a mean of 22 months. At follow-up, patients with plaques 1–3.9 mm thick (*n *= 45, group I) had an incidence of stroke, peripheral embolism, or death of 8.8% compared with 24.0% in patients with plaques ≥ 4 mm thick (*n* = 50, group II) and 39.2% in those with mobile components irrespective of plaque thickness (*n* = 34, group III) (*P* = 0.007). When the analysis was limited to mortality, significantly higher rates were obtained in patients with documented mobile plaque components (*P* = 0.049).

Cohen *et al.* [50] followed 334 patients ≥ 60 years of age which had a TEE immediately after a stroke for 2 to 4 years. In this study, patients with plaque thickness ≥ 4 mm without echocardiographically detectable calcification had a higher incidence of vascular events, reaching a RR of 10.3. The same findings were obtained in a study of coronary artery bypass graft surgery [51]. Hypoechoic plaques were associated with an increased incidence of perioperative stroke and embolic events. Possibly hypoechoic plaques (not calcified) have high lipid content, would be more easily fissured, and generation of superimposed thrombosis. In a histological study, it was found that plaques associated with thrombi were those with its highest volume proportion occupied by extracellular lipids, and with greater numbers of monocytes and macrophages at the level of the capsule [52].

All these data demonstrate a strong independent association between ischemic stroke and aortic arch atheroma located proximal to the ostium of the left subclavian artery, which is very suggestive of causality. This association is particularly evident in patients with stroke of unknown etiology, which also supports the view of causality. Plaque morphology is a powerful marker of embolic risk, being particularly high in the thickest plaques, when moving components are detectable, and possibly in the presence of ulceration and hypoechogenic echographic characteristics.

### Prospective Follow-Up Studies of Cerebral Infarction of Unknown Etiology

In a Spanish study, 1840 consecutive patients with a first-ever stroke were evaluated through a diagnostic protocol restrictive for TEE practice [53], and in 248 cases (13,5%) it was not possible to establish a definite etiologic diagnosis. This subgroup of patients received aspirin 300 mg/day over 1-year. TEE in association with all diagnostic work-up studies were repeated in 20 (8.1%) patients who developed a new arterial ischemic episode at follow-up (recurrent stroke in 13, transient cerebral ischemia in 3). All new events occurred within the first 4 months of follow-up. Except for TEE the remaining diagnostic studies were unrevealing. TEE showed complex plaques proximal to the ostium of the left subclavian artery in 15 of the 20 patients (75%) (10 cases with presence of mobiles components; 5 cases with both ≥ 4 mm plaque thickness plus plaque ulceration). On the other hand, the control group of the FSAPS population [49] (in which TEE was performed at baseline in all the 331 stroke patients included) was similar to the Spanish population with regard to age (75.7 vs 75.4 years), gender distribution, and prevalence of major cardiovascular risk factors (hypertension, hyperlipidemia, diabetes, smoking, atrial fibrillation, and history of myocardial infarction and peripheral vascular disease). Both studies used the same criteria for classifying patients as stroke of unknown etiology (no etiological diagnosis regardless of the presence or absence of aortic plaques), treated these patients with antiplatelet agents, and reported a similar incidence of recurrence of stroke and arterial embolism during follow-up. The Spanish patients with recurrent cerebral infarction of unknown etiology had a prevalence of complex aortic arch plaques of 82.4% compared with 21.1 % in the FSAPS patients with cerebral infarction of uncertain etiology treated with antiplatelet drugs who had no recurrence of stroke (*P* < 0.0001). These data also suggest an etiological role for complex aortic plaques and stroke especially when an identifiable cause is lacking, and in the case of embolic recurrence during treatment with aspirin. This observation seems particularly relevant for health care systems with accessibility limitations to TEE, which occurs especially in the context of geriatric stroke patients.

## IMAGING TECHNIQUES

**Fig. (1):**
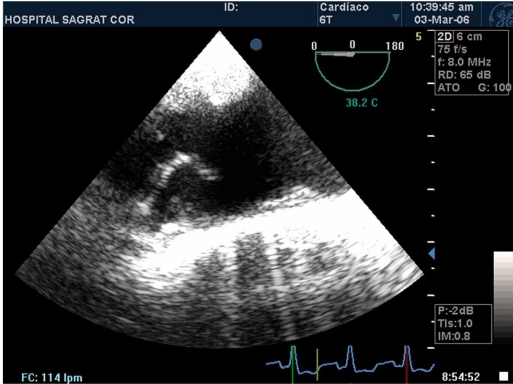
Transesophageal echocardiogram showing an aortic arch complex plaque with a mobile component.

TTE allows visualization of the aortic root and proximal ascending aorta. In some patients transcutaneous harmonic imaging from the suprasternal windows can reliably visualize protruding aortic arch atheromas, representing an excellent screening test and providing complementary views of regions which may be blind for TEE [54]. However, TEE is more accurate than TTE for detection of aortic arch atheromas and its mobile components (Fig. ** 1**), measuring plaque thickness and ulceration, and to define qualitatively its echogenicity, including the presence of calcification [55].

Magnetic resonance imaging (MRI) can provide important information on the characteristics of the atheromatous plaque and its component tissues, including the presence of calcification, fibrocellular tissue, lipid composition, and presence of thrombus [56, 57]. Stability of the plaque depends on the size of the lipid core, the thickness of the fibrous capsule, and the presence of inflammation, MRI offering the ability to provide valuable information on these three parameters [58]. However, in a comparative study with TEE, MRI underestimated the thickness of the plaques located at the aortic arch probably because of the difficulty in accurately defining its external limit in the angio-MRI [59]. However, this technique is expensive and not feasible in critically ill patients with stroke and neurologically unstable clinical circumstances, has frequent contraindications (prostheses, pacemakers, defibrillators, claustrophobia), and is less reliable than TEE for detection of mobile thrombi (which is the most powerful predictor of embolism). Therefore, MRI should not be considered a suitable technique for the routine study of these patients.

Computed tomography (CT) scanning is useful in evaluating the aorta and its major branches. The high-resolution helical CT allows detection of protruding aortic plaques and would be of particular interest in the study of areas not visualized by TEE (e.g., the distal ascending aorta). In two studies [60, 61], CT detected 94% and 95% of the plaques documented by TEE being also able to reliably assess plaque thickness. Finally, the combination of CT with positron emitting tomography (PET) allows fluorodeoxyglucose uptake in aortic atherosclerotic plaques to identify unstable plaques [62, 63].

In any case, the cost, complexity, technical problems, and limited accessibility to MRI and CT determine that TEE has to be considered as the technique of choice (gold standard) in the diagnosis of atherosclerotic plaques of the aortic arch as well as in the characterization of their morphological characteristics for embolic risk stratification. However, in some patients with good transcutaneous suprasternal windows, TTE may be sufficient to diagnose and classify the embolic risk, and in this case it contributes with the advantages of simplicity, safety, low-cost, and the possibility to be made at the patient’s bedside in cases of acute cerebral infarction and unstable neurological conditions.

## COMPLEX AORTIC ARCH TREATMENT IN STROKE PATIENTS

### Surgical 

There have been described isolated cases of surgical aortic arch endarterectomy in relatively young patients with complex aortic plaques and recurrent embolism despite anticoagulation [40, 41, 64]. Arko *et al.* [65] published a series of 23 patients with arterial embolism and aortic arch plaques with mobile components treated with surgical endarterectomy followed by warfarin; in 18 months of follow-up no new vascular events occurred and the mobile components had disappeared in all 6 patients who underwent a control TEE. However, in large clinical series it has been shown that aortic arch surgical endarterectomy is associated with an increased risk of extensive intraoperative cerebral infarction [66].

Currently, there are no data supporting the widespread introduction of surgical endarterectomy of the aortic arch in these patients, but this technique should be restricted to patients with severe recurrent arterial embolism unresponsive to anticoagulation and other supportive medical therapies.

### Treatment of Hypercholesterolemia

In patients with carotid or aortic arch atheromatosis, simvastatin reduces vascular wall thickness increasing the luminal area [67]. The Heart Protection Study [68] showed that simvastatin was effective in secondary prevention of vascular events in stroke patients even when plasma cholesterol levels were in the "normal" range. In accordance, the Cholesterol and Recurrent Events study (CARE) [69] included elderly patients with myocardial infarction and “normal” cholesterol levels, showing that treatment with pravastatin was associated with a clinically significant reduction in the risk of stroke and coronary events. 

To date there are no randomized studies on the effect of statins in patients with complex atherosclerotic aortic plaques. The main information available comes from an observational study of 519 patients with complex aortic arch plaques diagnosed by TEE, which documented an incidence of embolic events of 21% during the follow-up [70]. Multivariate analysis showed that administration of a statin had an independent protective effect on embolic events with a RR reduction of 59% (*P* = 0.0001). A study controlled by MRI [71] showed regression of plaque thickness during statin therapy, and the intensity of this effect was significantly associated with the level of decrease in serum levels of LDL-cholesterol. Two further studies confirmed regression of plaque thickness during statin therapy; in one of them [72], this effect was only associated with the LDL-cholesterol levels achieved, but in the other [73] regression was associated both with the level of LDL-cholesterol achieved and with the dose of statin administered.

Therefore, it seems very likely that statins reduce the risk of stroke in patients with complex plaques at the aortic arch. This could be related to its effects on plaque regression, plaque stabilization by reducing their lipid content, inhibition of inflammatory patterns in the lesion, and possible effects on coagulation. Consequently, statins should be administered to all stroke patients with complex aortic arch plaques independently of their plasma cholesterol levels, since part of the preventive benefits may be associated with the pleitropic effects of these compounds.

### Treatment of Hypertension

There are no available data on the effects of antihypertensive therapy on the clinical course or potential regression of complex aortic arch plaques. Like the case of cholesterol, in the PROGRESS study [74] the association of indapamide and perindopril reduced blood pressure and was effective in the secondary prevention of stroke even in patients whose baseline blood pressure was within the "normal" range. Although there are no randomized studies, hypertensive stroke patients with complex aortic arch plaques are at high risk of recurrent ischemic stroke and, therefore, both hypertension and other concomitant cardiovascular risk factors should be adequately treated.

### Antithrombotic and Anticoagulant Treatment

It has been shown that moving components superimposed to plaques generally correspond to thrombus [35, 40-44], and that this is the greatest risk factor for recurrent embolism [37, 53]. Accordingly, it seems logical to prescribe anticoagulation to prevent further ischemic events. Although there are no randomized studies on anticoagulation in stroke patients with high risk aortic arch complex plaques, this treatment was beneficial in two major observational studies. Dressler *et al.* [75] reported a series of 31 patients with complex aortic arch plaques and mobile components detected by TEE in which anticoagulation reduced the incidence of vascular events. At follow-up, stroke occurred in 3 of 11 patients not treated with anticoagulants as compared with none in the 20 patients treated with warfarin). Ferrari *et al.* [37] in a non-randomized observational study of 129 patients with aortic arch atherosclerotic plaques, which were detected by TEE performed to examine potential embolic sources, anticoagulation was associated with a significant reduction in embolic events (no emboli in 27 patients anticoagulated as compared with 5 strokes in 23 patients treated with platelet antiagregants). Additionally, anticoagulation reduced mortality in the subgroup of patients with mobile plaque components. However, all these data must be interpreted with caution because both aforementioned studies were observational and retrospective and the study population was small.

It has been shown that treatment with aspirin reduces the risk of recurrent stroke. The meta-analysis of the Antiplatelet Trialists Collaboration [76] showed that treatment with aspirin during 3 years was associated with a risk reduction of vascular events from 22% to 18%. However, in some patients with stroke and complex aortic arch plaques (especially when mobile components are seen), antiplatelet therapy with aspirin alone may be insufficient [37, 53]. In CAPRIE study [77], clopidogrel was superior to aspirin in patients with clinical manifestations of atherothrombotic disease, and the effect was particularly evident in some high risk subgroups. The CHARISMA study [78] evaluated the effect of adding clopidogrel to standard treatment with low-dose aspirin, and a modest beneficial effect (in the limit of significance) for dual antiplatelet therapy was only detected when analysis was restricted to the subgroup of 12,153 patients with already established cardiovascular disease (7.9% vs 6.9%; RR reduction of vascular events of 12.5%, 95% CI 0.77-0.998, *P* = 0.046). The MATCH study [79] compared the association of aspirin plus clopidogrel versus clopidogrel alone in patients with recent ischemic stroke or transient ischemic events, and no significant differences in the incidence of vascular events were found. In patients with stroke and complex aortic arch atherosclerotic plaques, antiplatelet therapy that seems to be more attractive is the association of aspirin and clopidogrel but in the absence of randomized prospective studies its effect should be considered uncertain and its possible use debatable.

## CONCLUDING REMARKS

In patients with ischemic cerebral infarction in whom standard non-invasive studies cannot identify any etiology, TEE should be performed to rule out the presence of complex aortic arch plaques especially in patients with cardiovascular risk factors. There is a high risk of short-term further embolic events when complex aortic arch plaques are found (≥ 4 mm thick, or presence of mobile components) but an increased risk is also present in case of ulcerated lesions and hypoechogenic plaques (which probably represent high lipid content). In these patients at high risk, in addition to treatment with statins and correction of the potential cardiovascular risk factors, anticoagulation or dual antiplatelet therapy with aspirin and clopidogrel should be considered. In the absence of randomized trials comparing the antithrombotic efficacy of these treatments, the limited data available from non-randomized retrospective studies support anticoagulation especially in the case of plaques with mobile components. Data provided in the near future by the ongoing ARCH international study [80] in which patients with ischemic stroke or peripheral arterial embolism and complex aortic arch plaques are randomized to treatment with warfarin or the combination of aspirin and clopidogrel will contribute to find final answers to all these questions. In particular, analysis in a pre-determined subset of patients with mobile atheroma will establish whether a more intensive antithrombotic treatment is required for these patients.

## Figures and Tables

**Table 1:** Data of Main Studies of Aortic Arch Atheroma in Stroke Patients or Patients Undergoing Transesophageal Echocardiography (TEE) Published in the Literature

Studies	Population	Definition of Complex Plaque	N(Cases/Controls)	Association with Clinical Events After Adjustement
**Post-Mortem**				
Amarenco *et al*. [21]	Neuologic disease	Ulcerated plaque	500	Yes
Khatibzadeh *et al*. [22]	Unselected	Ulceration, mural thrombus	120	Yes

**Case Series**				
Tunick and Kronzon[18]	TIA or stroke	Mobile	3	...
Davila-Roman *et al*. [9]	Cardiac surgery	> 4 mm, ulceration, mobile	1.200	Yes
Pop *et al*. [23]	TIA or stroke	Descriptive	72	...
Fasseas *et al*. [24]	Stroke	> 4 mm, ulceration, mobile	64	...
Toyoda *et al*. [25]	Embolic stroke	> 3 mm, irregular	62	...
Horowitz *et al*. [26]	TIA or stroke	Mobile	183	...
Mitush *et al*. [31]	Unselected TEE	> 5 mm, mobile	375	Yes

**Retrospective Case-Control Studies**				
Tunick *et al*. [27]	Stroke or embolism	> 5 mm	244 (122 / 122)	Yes
Di Tullio [29]	Stroke	> 5 mm, ulcerated or mobile	220	...
Di Tullio [30]	Stroke	> 5 mm, ulcerated or mobile	304 (152 / 152)	Yes
Stone *et al*. [32]	Stroke	> 2 mm or ulcerated	106 (49 / 57)	...
Nakayama *et al*. [34]	Stroke	Protruding, ulceration	80 (45 / 35)	...
Matsumura *et al*. [33]	Stroke	> 4 mm, mobile	451 (50 / 401)	...
Kazui *et al*. [38]	Stroke	> 5 mm	264 (62 / 202)	Yes

**Prospective Case-Control Studies**				
Jones *et al*. [28]	TIA or stroke	> 5 mm, ulcerated or mobile	417 (215 / 202)	Yes
Amarenco *et al*. [46]	Stroke	> 4 mm, ulcerated or mobile	500 (250 / 250)	Yes

**Prospective Follow-Up Studies**				
Ferrari *et al*. [37]	Unselected TEE	> 4 mm, mobile	129	Yes
Tunick *et al*. [47]	Unselected TEE	> 5 mm	84 (42 / 42)	Yes
FAPS group [49]	Stroke	> 4 mm	331 (45 / 286)	Yes
Mitush *et al*. [48]	Unselected TEE	> 5 mm, mobile	183 (47 / 136)	Yes
Pujadas *et al*. [53]	Stroke unknown cause	> 4 mm, mobile	350 (248 / 102)	

TIA: transient ischemic attack.
